# Screening for dual-target enzyme inhibitors derived from *Gardenia jasminoides* roots, a medicinal and edible plant: investigation of their potential anti-dementia activity

**DOI:** 10.3389/fnut.2025.1682948

**Published:** 2025-11-20

**Authors:** Xuanlin Liu, Yang Zhou, Rui Meng, Jingyi Han, Ming Chen, Xiangjie Bo, Sainan Li, Daqian Song, Yuchi Zhang

**Affiliations:** 1 Central Laboratory, Changchun Normal University, Changchun, China; 2 Department of Chemistry, Northeast Normal University, Changchun, Jilin, China; 3 College of Chemistry, Jilin Province Research Center for Engineering and Technology of Spectral Analytical Instruments, Jilin University, Changchun, China

**Keywords:** *Gardenia jasminoides* root, 5-lipoxygenase, acetylcholinesterase, activity screening, offline 2D-CCC

## Abstract

**Introduction:**

*Gardenia jasminoides* root (*GJR*) is a traditional Chinese plant valued for its dual functions as both a medicinal herb and an edible resource. Alzheimer's disease (AD) is an irreversible, fatal neurodegenerative disorder in the elderly, and current treatments mainly rely on single-target acetylcholinesterase (AChE) inhibitors with limited effects on disease progression. Thus, there is an urgent need to develop dual-target inhibitors that regulate inflammation (via 5-lipoxygenase, 5-LOX) and improve cholinergic dysfunction (via AChE).

**Methods:**

To efficiently and accurately screen active compounds, receptor-ligand affinity ultrafiltration coupled with enzyme kinetics was used for rapid identification and characterization. Biochemical assays validated the inhibitory activities and mechanisms of the compounds, while molecular docking and molecular dynamics simulations evaluated target binding affinity and stability at the atomic level. An offline two-dimensional chromatographic method was developed to overcome the limitations of conventional countercurrent chromatography, enhancing peak capacity, and separation efficiency.

**Results:**

Seven active compounds were successfully isolated and identified from *GJR*, including Shanziside, Deacetylasperulosidic acid methyl ester, Gardoside, Shanzhiside methyl ester, Mussaenoside acid, Eleutheroside E, and 5-Hydroxy-3′,4′-dimethoxyflavone. These compounds exhibit potential dual-target inhibitory effects on 5-LOX and AChE, laying the foundation for anti-AD research.

**Discussion:**

This study integrates advanced screening, optimized extraction, and rigorous bioactivity assessment to elucidate the active components of *GJR* and their anti-AD potential. The developed methodology addresses the shortcomings of single-target drug development and provides valuable insights for the development of dual-target inhibitors and the advancement of plant-based food preparation technologies.

## 1 Introduction

Achieving optimal therapeutic outcomes through pharmacological intervention alone remains a significant challenge in modern health management. Dietary therapy, as a natural and holistic approach, plays a complementary role by modulating physiological functions and promoting overall wellbeing (1). Certain foods not only supply essential nutrients but also exhibit pharmacological effects comparable to those of conventional drugs, exemplifying the principle of food-drug homology. *Gardenia jasminoides* root (*GJR*) is used to treat icterohepatitis, renal edema, high fever with chills, hemorrhage, and hematemesis. Additionally, it shows potential for further development as a dual-purpose crop for both medicine and food (2).

Alzheimer's disease (AD) is a progressive neurological disorder characterized by the impairment of judgment, thinking ability, orientation, and cognitive function (3–6). It is projected that by 2050, one in every 85 individuals globally will be affected by AD, attributable to the rising incidence of the disease within the aging population (7, 8). The disease is characterized by acetylcholine (ACh) deficiency, amyloid-beta (Aβ) plaque accumulation, neurofibrillary tangle (NFT) progression, and oxidative stress-induced nerve injury (9). Accordingly, current therapeutic strategies emphasize the development of cholinesterase inhibitors, antioxidants, anti-amyloid agents, and anti-inflammatory drugs (10–12). Among the molecular targets associated with AD, 5-lipoxygenase (5-LOX) plays a pivotal role in cellular metabolism and is strongly implicated in inflammation, cancer, and neurodegenerative diseases (13). Inhibiting 5-LOX activation reduces neuroinflammation and helps alleviate cognitive impairment in AD. This finding provides insights for developing new anti-AD drugs. Similarly, acetylcholinesterase (AChE) hydrolyzes ACh, thereby reducing its synaptic availability and contributing to the impairment of cholinergic signaling in AD (14). AChE hydrolyzes acetylcholine, impairing neural signaling. Elevated AChE activity reduces acetylcholine levels, promoting AD pathogenesis. Inhibiting AChE is a key strategy for AD treatment (15, 16). Given the critical roles of both 5-LOX and AChE in AD pathogenesis, a multi-target drug discovery strategy offers considerable advantages over conventional single-target approaches.

In this study, we established a comprehensive methodology to screen and evaluate bioactive compounds from *GJR*. Affinity ultrafiltration (AUF) was employed to selectively isolate small-molecule ligands with high binding affinity to 5-LOX and AChE, followed by structural identification via high-performance liquid chromatography–mass spectrometry (HPLC-MS) (17). Enzyme inhibition kinetics were subsequently analyzed to classify the type of inhibition and eliminate false-positive candidates (18). Enzyme kinetics provide crucial insights into the reaction rates and underlying mechanisms that govern enzyme–substrate interactions (19). Finally, the catalytic mechanism of the enzymes was further investigated using molecular dynamics simulations and the conformational relationships between molecules to guarantee more accurate results for the inhibition experiments of *GJR* (20). Based on the results obtained from docking, potential proteins and specific compounds with targeted effects were identified to investigate the interaction between active ingredients and disease-related target proteins, as well as their stable binding ability (21–23). These computational techniques enabled us to identify key protein–compound interactions and predict binding affinities at the atomic level. To ensure a rigorous assessment of target activity, both experimental and *in silico* approaches were integrated to evaluate three specific mechanistic aspects of *GJR*-derived compounds (24).

**Figure 1:**
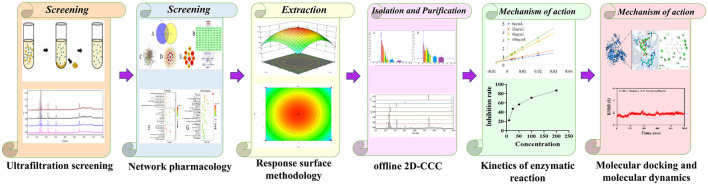
Workflow diagram for the screening and isolation of 5-LOX and AChE inhibitors from *Gardenia jasminoides* root.

Overall, we present an efficient and robust strategy for the rapid identification, extraction, and characterization of dual-target enzyme inhibitors from *GJR*. This approach not only addresses the limitations of single-target drug development but also enhances the applicability of *GJR* components in anti-inflammatory and neuroprotective therapies. In addition, molecular dynamics simulations were used to visualize and validate the interactions between active compounds and their respective targets, namely 5-LOX and AChE, thereby corroborating the findings of the AUF-HPLC-MS analysis (Figure 1).

## 2 Materials and methods

### 2.1 Apparatus

Offline two-dimensional counter-current chromatography (2D-CCC) was conducted using a TBE300B Spectrum HSCCC system (TAUTO, Shanghai, China). The system was equipped with a 2545 Quaternary Gradient Module pump, a Waters 2489 UV/Vis detector, and a Fraction Collector III. High-performance liquid chromatography (HPLC) analysis was carried out on a Waters 2695 system coupled with a Waters 2998 diode array detector. Separation was performed using an analytical SunFire™ C18 column (5.0 μm, 250 × 4.6 mm; Waters Corporation, USA).

### 2.2 Experimental materials

Samples of *Gardenia jasminoides root* were independently extracted and authenticated by the Changchun branch of Beijing Tong Ren Tang-one of China's leading medicinal companies-and further verified by Prof. Yuchi Zhang. Acetylcholinesterase (AChE, EC 3.2.1.20) was extracted from fly heads. 5-Lipoxygenase (5-LOX) and phosphate-buffered saline (PBS) were purchased from Fluka (Buchs, Switzerland). For the experimental procedures, a Microcon YM-100 ultrafiltration device (molecular weight cutoff: 100 kDa) was used to facilitate efficient and cost-effective separation. HPLC-grade acetonitrile was obtained from Thermo Fisher Scientific (Beijing, China). All other reagents and analytical-grade solvents were sourced from Guoyaohushi Corporation (Shanghai, China). Milli-Q^®^ water (specific resistivity: 18.2 MΩ·cm) was supplied by Millipore (Boston, MA, USA) and pretreated using a purification system prior to use. All experimental materials were handled and prepared in accordance with standard laboratory protocols.

### 2.3 Screening of enzyme inhibitors

A total of 300 g of *GJR* was accurately weighed, dried, and ground into a fine powder using a mechanical grinder. The powdered material was extracted with 60% ethanol (v/v) at a solvent-to-solid ratio of 20:1 (v/v). Each extraction cycle involved ultrasonication for 1 h and was performed twice to ensure exhaustive extraction. The combined extracts were concentrated to dryness by rotary evaporation under reduced pressure, yielding 6.75 g of crude extract. A 50 mg portion of the crude extract was accurately weighed, dissolved in 50% methanol–water (v/v), and diluted to a final concentration of 50 mg/ml to obtain a stock solution for subsequent experiments. All procedures were conducted under controlled laboratory conditions to ensure consistency and reproducibility.

The screening assay consisted of two groups: a blank control and an experimental group, both prepared in phosphate-buffered saline (PBS). In the experimental group, 10.0 μl of the sample solution was mixed with 90.0 μl of either 5-lipoxygenase (5-LOX) and acetylcholinesterase (AChE) at final concentrations of 0.5, 1.0, or 2.0 U/ml, along with 100 μl of PBS (pH 7.4), resulting in a total volume of 200 μl. The mixture was incubated at 37 °C for 30 min and subsequently filtered through a 100 kDa ultrafiltration membrane by centrifugation at high speed for 10 min. The supernatant was discarded, and the membrane was washed three times with PBS to remove free or non-specifically bound compounds. Then, 150 μl of 50% methanol–water was added to denature the proteins and release the bound small molecules. For the blank control, the enzyme solution was replaced with an equal volume of PBS, while all other experimental conditions remained the same. The resulting eluate was analyzed by HPLC. The binding degree (BD) was used to quantify the interaction between compounds and the target enzymes. It was calculated based on the increase in chromatographic peak area of enzyme-bound compounds, using the following formula:


$$\begin{array}{l} BD\left(\%\right)=\left(A_{1}-A_{2}\right)/A_{2}\times \;100 \end{array}$$

where A_1_ represents the peak area of the compound in the enzyme-binding group, and A_2_ represents the peak area in the blank control group.

### 2.4 Disease target prediction methods and conditions in network pharmacology

The SMILES (Simplified Molecular Input Line Entry System) strings of the *Gardenia jasminoides root* (*GJR*) components were retrieved from the PubChem database. Their potential protein targets were predicted using the Swiss Target Prediction platform. Only candidate targets with a prediction probability greater than zero were retained. The predicted targets from all components were then consolidated, and duplicate entries were removed to generate a comprehensive list of potential GJR-related targets. To identify disease-relevant genes, Alzheimer's disease-related targets were retrieved by querying the GeneCards and OMIM (Online Mendelian Inheritance in Man) databases using the keyword “Alzheimer's disease.”

The predicted targets of the *GJR* components were intersected with Alzheimer's disease-related genes to identify shared targets. A Venn diagram was generated to visualize the overlapping genes between the compound-related and disease-related target sets. Subsequently, Cytoscape software (version 3.8.2) was employed to construct a “compound–target–disease” interaction network, illustrating the multi-target relationships between *GJR* components and AD-related genes.

To further explore the protein–protein interactions (PPIs) associated with the therapeutic effects of GJR, the overlapping genes between GJR-related and Alzheimer's disease-related targets were uploaded to the STRING database (https://string-db.org/). The organism was set to “Homo sapiens,” and the minimum required interaction score was set to 0.4 to ensure data reliability. All other parameters were maintained at their default values. The PPI network data were exported in TSV format and subsequently imported into Cytoscape (version 3.8.2) for network visualization and analysis. Network topology parameters, including Degree, Closeness, and Betweenness centralities, were calculated using the CentiScape 2.1 plugin (Cytoscape → Apps → CentiScape 2.1 → Start). Node size and color were adjusted to visually represent the Degree value of each target gene, with larger nodes indicating higher connectivity within the network. Finally, the CytoHubba plugin was employed to identify key nodes in the network. The top 10 targets ranked by Degree were screened as core targets for subsequent analysis, forming the basis of the final protein–protein interaction network.

The overlap genes between drugs and diseases were uploaded to the DAVID database (https://davidbioinformatics.nih.gov/). The gene identifiers were set as OFFICIAL_GENE_SYMBOL, and the species was specified as Homo sapiens. The DAVID 6.8 Gene Ontology (GO) tool was utilized to analyze the Biological Process (BP), Cellular Component (CC), and Molecular Function (MF) categories, thereby annotating the roles of target proteins in the gene functions associated with Gardeniae fructus. To elucidate the role of signaling pathways associated with the targets of *GJR* in disease mechanisms, KEGG pathway enrichment analysis was conducted. The top 10 Gene Ontology (GO) functions (Biological Process, Cellular Component, Molecular Function) and the top 30 KEGG pathways were selected as the primary gene function enrichment processes and signaling pathways of *Gardeniae jasminoides* root. This selection aimed to predict the underlying mechanisms of *Gardeniae jasminoides* root in disease treatment.

### 2.5 Extraction process optimization using RSM

Ethanol-water solutions were employed as extraction solvents, and extraction yield was used as the evaluation index to systematically screen various extraction conditions. Ultrasound-assisted extraction parameters were optimized using response surface methodology (RSM). A Central Composite Design (CCD) was applied to assess the effects of four independent variables, each tested at three levels. Multivariate regression analysis was performed using Design-Expert software (version 8.0.6), resulting in a predictive model correlating total yield with extraction time (A), number of extraction cycles (B), ethanol concentration (C), and liquid-to-solid ratio (D). Experimental errors were assessed using center point replicates, with factor levels coded as +1 (high), 0 (medium), and −1 (low).

### 2.6 Determination of HSCCC partition coefficient (K) and 2D-CCC

Approximately 200 mg of sample was accurately weighed, transferred to a test tube, and mixed with 4.0 ml of a biphasic solvent system. After vigorous shaking and equilibration, the two phases were separated. Each phase was evaporated to dryness in a water bath, and the resulting residues were dissolved in methanol for HPLC analysis. The partition coefficient (K) was defined as the ratio of peak areas of the compound in the upper phase (AU) and the lower phase (AL), calculated as: *K* = A_U_/A_L_. Based on the results, a two-phase solvent system composed of n-hexane/ethyl acetate/ethanol/water (1.5:12.0:3.0:10.0, v/v/v/v) was selected for its efficiency in rapidly separating and purifying the seven target compounds.

A two-phase solvent system consisting of *n*-hexane/ethyl acetate/ethanol/water (1.5:12.0:3.0:10.0, v/v/v/v) was prepared for the first and second solvent systems during offline 2D-CCC separation. The system was equilibrated at room temperature by shaking in a separating funnel. Before use, the upper and lower phases were separated and degassed by sonication for 20 min.

For the initial CCC separation, 300 mg of crude extract was dissolved in 8.0 ml of a 1:1 mixture of mobile and stationary phases to prepare the sample solution. During the first-dimensional CCC, the eluate following the solvent peak was collected and dried in a water bath. The dried solutes were then redissolved in 8.0 ml of mobile phase to prepare sample solutions for offline two-dimensional CCC separation. During the first-dimensional CCC, the eluent following the solvent peak was collected, evaporated to dryness in a water bath, and re-dissolved in 8.0 ml of mobile phase to serve as the sample for the second-dimensional separation.

The CCC separation was conducted by first filling the column with the stationary phase (upper phase) using a constant flow pump at 35 ml/min, with the column maintained at 25 °C. Once partially filled, the mobile phase (lower phase) was introduced at 3.0 ml/min. The CCC unit was rotated at 850 rpm during column filling. Wastewater was collected to monitor the remaining stationary phase. When a clear mobile phase emerged and hydrodynamic equilibrium was achieved, the sample solution was injected. A UV detector at 280 nm monitored the process, with peak data automatically recorded by a scoring system. The second dimension followed the same procedure.

### 2.7 Enzyme inhibition mechanism

All reagents were prepared with 0.05 mol/L phosphate buffer solution at pH 6.8. 0.5 mg of Mussaenoside acid was dissolved in the phosphate buffer solution to prepare a 5.0 × 10^−3^ mol/L sample solution for later use. Before use, it was diluted to 0, 5, 10, 15, and 20 × 10^−5^ mol/L. 5-LOX and linoleic acid were, respectively prepared at concentrations of 2.08 × 10^−7^ and 5.0 × 10^−4^ mol/L. AChE and iodinated thioacetylcholine were prepared using the same method. The enzyme concentrations were diluted to 0.5, 1.0, 1.5, 2.0, and 2.5 × 10^−7^ mol/L, and the substrate concentrations were diluted to 0.5, 1.0, 1.5, 2.0, and 2.5 × 10^−4^ mol/L. Galantamine was prepared at a concentration of 4.0 × 10^−3^ mol/L.

Galantamine served as the positive control. In a PBS buffer system (0.05 mol/L, pH 6.8), the concentration of 5-LOX was maintained at a constant level (2.08 × 10^−7^ mol/L), and various concentrations of Mussaenoside acid were introduced. The mixture was incubated for 10 min at 37 °C in a thermostatic water bath to form the enzyme reaction solution, followed by the addition of linoleic acid (5.0 × 10^−4^ mol/L) as the substrate. Prior to HPLC analysis, all test samples were filtered through a 0.45 μm microporous membrane, with each experimental group independently measured in triplicate. For the AChE assay, 0.1 ml of DTNB solution was included in the reaction mixture and incubated under the same conditions as those used in the 5-LOX experiment, while all other procedural steps remained identical. The control group was not supplemented with samples, while the blank group received an equal volume of buffer solution in place of the enzyme solution. The half-maximal inhibitory concentration (IC_50_) of Mussaenoside acid against 5-LOX and AChE was determined. Enzyme activity in the absence of any inhibitor was regarded as 100%. The IC_50_ value represents the concentration of Mussaenoside acid required to inhibit 50% of the enzymatic activity of 5-LOX and AChE.

Under identical conditions to those used in the enzyme activity assay, the substrate concentration was maintained at a constant level of 5.0 × 10^−4^ mol/L. Following the addition of varying concentrations of Mussaenoside acid, the corresponding enzymatic reaction rates were recorded. By analyzing the correlation between reaction rate and enzyme concentration, a kinetic curve was generated. Subsequent data interpretation was performed to determine the reversibility of Mussaenoside acid's inhibitory effects on 5-LOX and AChE.

Under identical experimental conditions, the concentrations of 5-LOX and AChE were maintained at 2.08 × 10^−7^ mol/L. The enzymatic reaction rates of Mussaenoside acid were measured across varying substrate concentrations. By applying the Lineweaver-Burk equation to plot the data, the inhibition modes of Mussaenoside acid against 5-LOX and AChE were identified, and the corresponding inhibition constants (K_*i*_) were calculated. Data analysis was carried out using Origin 8.0 software through one-way ANOVA, with each sample group subjected to three independent replicate experiments (*p* < 0.05).

### 2.8 Molecular docking and molecular dynamics simulations were performed to verify the active compounds

#### 2.8.1 Molecular docking analysis

Through the PubChem database (http://pubchem.ncbi.nlm.nih.gov/), the two-dimensional (2D) structures of small molecular ligands were retrieved. These 2D structures were subsequently imported into ChemOffice to generate three-dimensional (3D) structures, which were saved in mol2 format. Set the coordinates and dimensions of the grid box as follows: (X = 116.412, Y = 104.282, Z = −142.677), (X = 41.25, Y = 37.5, Z = 42.75). Molecular docking simulations were conducted using AutoDock Vina version 1.5.6 to investigate protein-ligand interactions. Prior to docking, Autodock tools were employed to process the protein and ligand structures, including protein hydrogenation, dehydration, ligand hydrogenation, and determination of torsion angles. Docking box coordinates were defined based on the target binding site, and the optimal conformation was identified by comparing the docking scores. Finally, the interactions between the test compounds and key residues were visualized in both two-dimensional (2D) and three-dimensional (3D) formats using PyMOL and Discovery Studio 2019 software. Generally, in the context of molecular interactions, when the binding energy is < −5.0 kcal/mol, it suggests that the two molecules exhibit favorable binding activity. A value below −7.0 kcal/mol indicates strong binding activity. Lower binding energy correlates with stronger binding activity, higher affinity, and more stable conformation.

#### 2.8.2 Molecular dynamics simulations

The complexes were simulated using 100 ns molecular dynamics (MD) with GROMACS 2022. The CHARMM36 force field (25) was employed for protein parameterization, while the ligand topology was generated based on GAFF2 force field parameters. Periodic boundary conditions were applied, and the protein-ligand complex was centered within a cubic box. The TIP3P water model was used to solvate the system, forming a water-filled cubic box with a minimum periodic boundary distance of 1.2 nm from the solute (26). Electrostatic interactions were treated using the particle mesh Ewald (PME) method, while non-bonded interactions were managed with the Verlet neighbor list algorithm. Subsequently, the system underwent 100,000 steps of equilibration in both the isothermal-isochoric (NVT) and isothermal-isobaric (NPT) ensembles. For these equilibration stages, a coupling constant of 0.1 ps was employed, and the total simulation duration was set to 100 ps. Van der Waals and Coulomb interactions were computed with a cutoff distance of 1.0 nm. Finally, the system was subjected to a 100 ns molecular dynamics simulation using Gromacs 2022 under constant temperature (310 K) and pressure (one bar) conditions.

## 3 Results

### 3.1 Screening and identification of enzyme inhibitors

**Figure 2:**
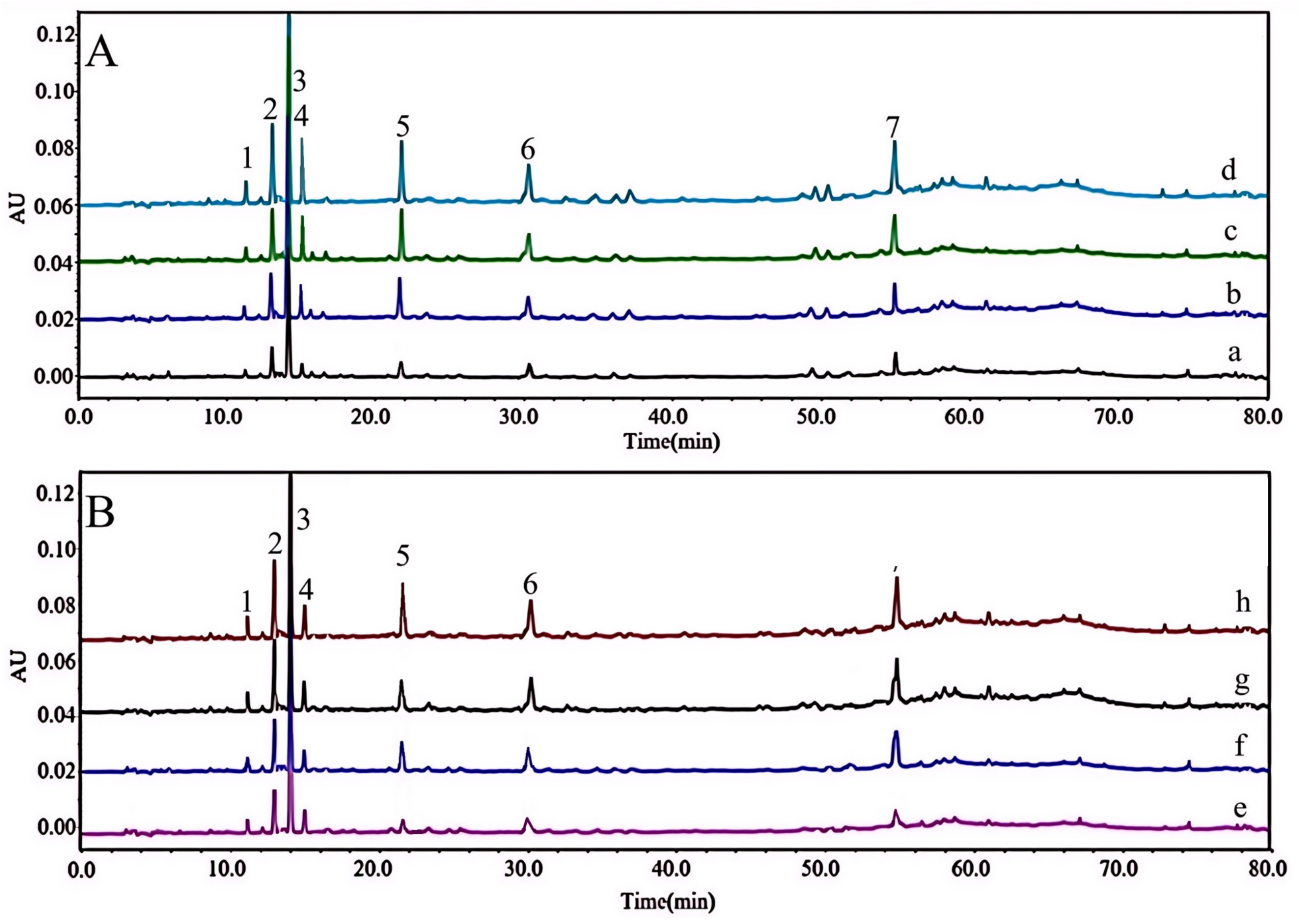
UF-HPLC of the extract obtained from *GJR* binding to different concentrations of 5-LOX **(A)** and AChE **(B)**: (a–d) 5-LOX concentrations of 0.0, 0.5, 1.0, and 2.0 U/ml; **(e–h)** AChE concentrations of 0.0, 0.5, 1.0, and 2.0 U/ml. (1): Shanziside, (2): Deacetylasperulosidic acid methyl ester, (3): Gardoside, (4): Shanzhiside methyl ester, (5): Mussaenoside acid, (6): Eleutheroside E, (7): 5-Hydroxy-3′,4′-dimethoxyflavone.

AUF-LC-MS analysis identified seven compounds that bind specifically to 5-LOX and AChE, exhibiting potential enzyme inhibitory effects (Figures 2A, B). The binding degree (BD) of each compound was calculated according to the following equation: BD (%) = (A_1_-A_2_)/A_2_ × 100 (27), where A_1_ denotes the peak area of the enzyme-bound compound, whereas A_2_ indicates the peak area of the free compound. The retention times (t_R_), mass spectrometry (MS) data, and binding affinity values for the seven compounds are summarized in Table 1.

**Table 1:** Chemical composition analysis and binding affinities of the extract obtained from *Gardenia jasminoides* root toward 5-LOX and AChE.

**Peak no**.	**t_R_/min**	**MS (m/z)**	**MS^2^**	**Identity**	**Formula**	**BD** ^**a**^			**BD** ^**b**^		
						**0.5 U/ml**	**1.0 U/ml**	**2.0 U/ml**	**0.5 U/ml**	**1.0 U/ml**	**2.0 U/ml**
1	11.27	391.1245	391.1257, 229.0710, 185.0811, 127.0411	Shanziside	C_16_H_24_O_11_	52.16	58.19	67.46	18.79	34.41	43.34
2	13.04	449.1298	449.1256, 403.1256, 241.0703, 127.0401	Deacetylasperulosidic acid methyl ester	C_17_H_24_O_11_	42.49	64.59	91.74	34.54	40.99	41.52
3	14.15	373.1144	373.1164, 211.0606, 149.0607, 59.0200	Gardoside	C_16_H_22_O_10_	51.18	62.85	71.81	26.11	33.46	39.15
4	15.08	405.1398	405.1401, 225.0810, 197.0755, 149.0612	Shanzhiside methyl ester	C_17_H_26_O_11_	58.75	78.44	98.64	21.37	40.69	69.22
5	21.75	375.1294	301.0561, 213.0752, 151.0744, 107.0512	Mussaenoside acid	C_16_H_24_O_10_	76.08	82.25	93.73	16.25	23.04	92.88
6	30.29	741.2617	706.0896, 579.2177, 417.1578, 181.0485	Eleutheroside E	C_34_H_46_O_18_	38.17	55.16	59.44	12.19	21.06	69.87
7	54.92	299.0927	299.0912, 283.0617, 241.0493, 185.0625	5-Hydroxy-3′,4′-dimethoxyflavone	C_17_H_16_O_5_	56.63	70.86	95.05	15.79	22.51	77.27

BD refers to binding degree of the target compounds with ^a^5-LOX and ^b^AChE.

The findings indicate that the inhibitory effects of the active constituents of *GJR* on 5-LOX and AChE markedly enhance as the enzyme concentration rises. At a concentration of 2.0 U/ml, each compound exhibits its strongest binding affinity. Notably, mussaenoside acid demonstrates a binding efficiency exceeding 90% with both enzymes at this concentration (Table 1).

Thus, the binding affinity of the active constituents in *GJR* to 5-LOX and AChE rises proportionally with increasing enzyme concentration. Mussaenoside acid demonstrates significant dual-target inhibitory effects, indicating promising potential for anti-AD activity.

### 3.2 Network pharmacology

#### 3.2.1 GJR active ingredients and disease-associated targets

**Figure 3:**
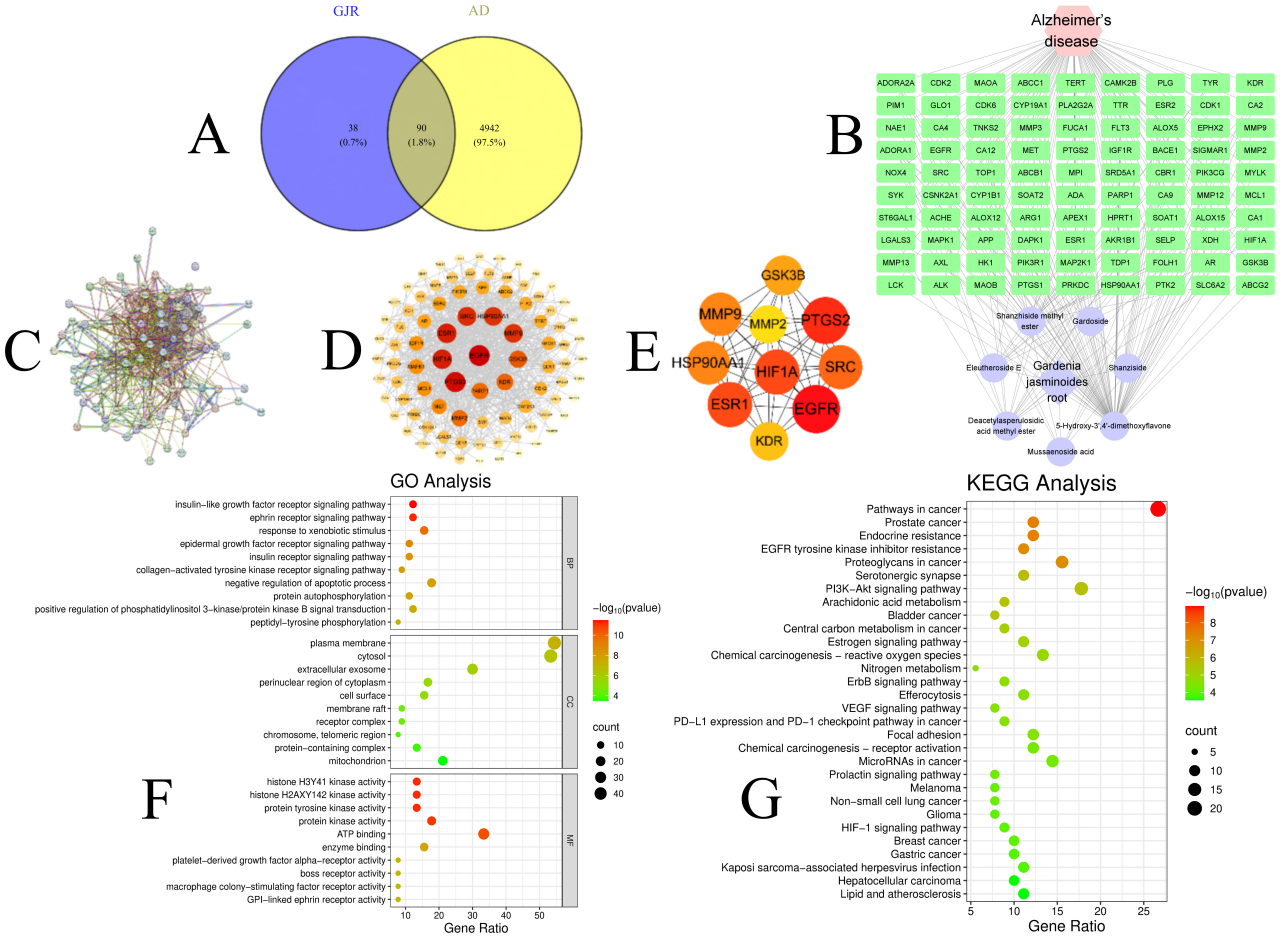
**(A)** Venn diagram; **(B)** Illustration of the GJR-AD Network Structure, circles represent targets, hexagons represent drugs, and diamonds represent diseases; **(C)** Target protein interaction (PPI) network at drug-disease intersection; **(D)** Cytoscape was used to analyze the drug-disease PPI network; **(E)** Core Targets; **(F)** GO enrichment analysis; **(G)** KEGG pathway enrichment analysis.

The candidate targets were screened from the SWISS database using a probability >0 as the criterion. After the removal of duplicate entries for each component target, a total of 128 unique targets were identified as potential targets of *GJR*. After integrating the GeneCards and OMIM databases using a relevance score threshold >5, 5,032 targets associated with AD were identified. Intersection analysis of drug targets and disease-related genes revealed 90 overlapping target genes, which represent the interaction targets through which GJR exerts its therapeutic effects (Figure 3A).

#### 3.2.2 Construction and analysis of the “GJR-AD” network

To gain deeper insights into the key constituents of *GJR*, their corresponding targets, and their potential links to Alzheimer's disease, the “*GJR*-AD” interaction network was established using Cytoscape (version 3.9.0; Figure 3B). By loading the preformatted “Network.xlsx” file into Cytoscape, the network mapping the relationships between *GJR* and AD was successfully generated. The topological properties of the network were systematically analyzed using the Network Analyzer plugin. Key parameters included an average degree of 7.540, a heterogeneity index of 2.820, a network density of 0.029, and a centrality value of 0.941, reflecting a well-connected and structurally significant interaction pattern. Nodes with higher degree values are considered central to the network topology, indicating their potential functional significance. The most highly connected active components include Mussaenoside acid (Degree = 137), Shanzhiside methyl ester (Degree = 78), and Deacetylasperulosidic acid methyl ester (Degree = 70), suggesting their pivotal roles in the pharmacological network. The therapeutic effects of *GJR* against AD are likely mediated by active compounds that exhibit a high number of interaction targets and strong connectivity within the network, fulfilling a central hub role in the pharmacological architecture. Furthermore, individual components with multi-target potential can concurrently modulate multiple biological targets, while several components may converge on a single target, indicating synergistic interactions. This evidence suggests that *GJR* exerts its influence on AD through a coordinated, multi-component, multi-target regulatory mechanism.

#### 3.2.3 PPI network analysis

To gain deeper insights into the molecular mechanisms underlying *GJR*'s therapeutic effects on AD, a protein–protein interaction (PPI) network was constructed to analyze the functional relationships among shared target genes. By intersecting all drug-related targets with AD-associated disease genes, a total of 90 overlapping target genes were identified as potential mediators of *GJR*'s pharmacological activity. These 90 common targets were subsequently uploaded to the STRING database (https://string-db.org/) to predict their protein–protein interactions, with the species restricted to Homo sapiens and the interaction confidence score set at 0.4. Core targets were then determined based on topological analysis, where genes exhibiting values above the mean for key network parameters—including Betweenness Centrality, Closeness Centrality, and Degree—were selected to represent central nodes in the network. Among these targets, the top 10 ranked by Degree centrality are HIF1A, PTGS2, ESR1, EGFR, HSP90AA1, MMP9, GSK3B, SRC, MMP2, and KDR. These ten genes represent high-connectivity hub nodes in the network and are likely to play critical roles in the pathogenesis of AD as key mediators of *GJR*'s therapeutic effects (Figures 3C–E).

#### 3.2.4 Biological function and gene pathway enrichment analysis of intersection targets

The main biological processes derived from GO and KEGG enrichment analyses help identify the functional roles of the target proteins. To investigate the involvement of intersection targets associated with AD in gene functions and signaling pathways, GO functional enrichment and KEGG pathway enrichment analyses were performed using R software. The intersection targets were analyzed via the “clusterProfiler” R package for GO enrichment. In the biological process (BP) category, significantly enriched terms included the insulin-like growth factor receptor signaling pathway, ephrin receptor signaling pathway, response to xenobiotic stimulus, epidermal growth factor receptor signaling pathway, and insulin receptor signaling pathway, indicating their potential regulatory roles in AD pathogenesis. The main molecular functions (MF) include histone H3Y41 kinase activity, histone H2AXY142 kinase activity, protein tyrosine kinase activity, protein kinase activity, and ATP binding. Cellular components (CC) mainly involve plasma membrane, cytosol, extracellular exosome, perinuclear region of cytoplasm, and cell surface. The analysis results reveal that potential targets are enriched in key biological processes, including inflammatory response and cell cycle regulation. These processes are closely associated with the pathogenesis of AD, indicating that *GJR* exerts its therapeutic effects on AD through modulation of multiple biological pathways (Figure 3F).

KEGG pathway enrichment analysis was conducted on the intersection targets of *GJR* and AD through the “Cluster Profiler” package of R language software. The results showed that there were a total of 167 signaling pathways intervened by *GJR* in AD (q value < 0.05), mainly involving Pathways in cancer, Prostate cancer, Endocrine resistance, and EGFR tyrosine kinase inhibitor resistance, Proteoglycans in cancer, Serotonergic synapse, PI3K-Akt signaling pathway, Arachidonic acid metabolism, Bladder cancer, Central carbon metabolism in cancer (Figure 3G).

### 3.3 Extraction and preparation of targeted compounds

Design Expert 8.0.6 was employed to develop the experimental design, using the total flavonoid yield as the response variable. The independent variables included extraction time (A), number of extraction (B), ethanol concentration (C), and liquid-solid ratio (D). The analysis of variance (ANOVA) results are presented in Table 2.

**Table 2:** Anova table for the regression model.

**Source**	**Sum of squares**	**df**	**Mean square**	***F*-value**	***p*-Value**	**Statistical significance**
Model	0.1112	14	0.0079	18.99	< 0.0001	Significant
A–A	0.0001	1	0.0001	0.3030	0.5907	
B–B	0.0003	1	0.0003	0.6473	0.4345	
C–C	0.0031	1	0.0031	7.50	0.0160	
D–D	0.0000	1	0.0000	0.0287	0.8679	
AB	0.0000	1	0.0000	0.0484	0.8290	
AC	0.0001	1	0.0001	0.2391	0.6324	
AD	0.0003	1	0.0003	0.7746	0.3936	
BC	0.0003	1	0.0003	0.7322	0.4066	
BD	0.0000	1	0.0000	0.0484	0.8290	
CD	0.0004	1	0.0004	0.9091	0.3565	
A^2^	0.0347	1	0.0347	82.89	< 0.0001	
B^2^	0.0527	1	0.0527	125.92	< 0.0001	
C^2^	0.0439	1	0.0439	104.87	< 0.0001	
D^2^	0.0346	1	0.0346	82.61	< 0.0001	
Residual	0.0059	14	0.0004			
Lack of fit	0.0054	10	0.0005	5.17	0.0638	Not significant
Pure error	0.0004	4	0.0001			
Cor total	0.1171	28				

The multiple quadratic regression of each factor was conducted by using analysis of variance, and the regression equation was obtained as follows: Y = +0.3898 + 0.0032*A* – 0.0048*B* – 0.0162*C* + 0.0010*D* – 0.0022*AB* + 0.0050*AC* + 0.0090*AD* – 0.0087*BC* + 0.0023*BD* – 0.0098*CD* – 0.0731*A*^2^ – 0.0901*B*^2^ – 0.0822*C*^2^ – 0.0730*D*^2^, where Y represents the calculated yield. Table 2 analysis shows the model is significant (*p* < 0.01) with nonsignificant lack of fit (*p* > 0.05). A^2^, B^2^, C^2^, D^2^ are significant. Thus, the model is ideal for predicting and analyzing extraction conditions. *R* is 0.9500, Adj *R*^2^ is 0.9000, explaining 90% of experiments. Pre *R*^2^ (0.7270) is close to Adj *R*^2^, and the signal–to–noise ratio is 13.734, indicating reliable predictions. If the SNR is >4, the model can be used to predict new observations with high confidence (28).

**Figure 4:**
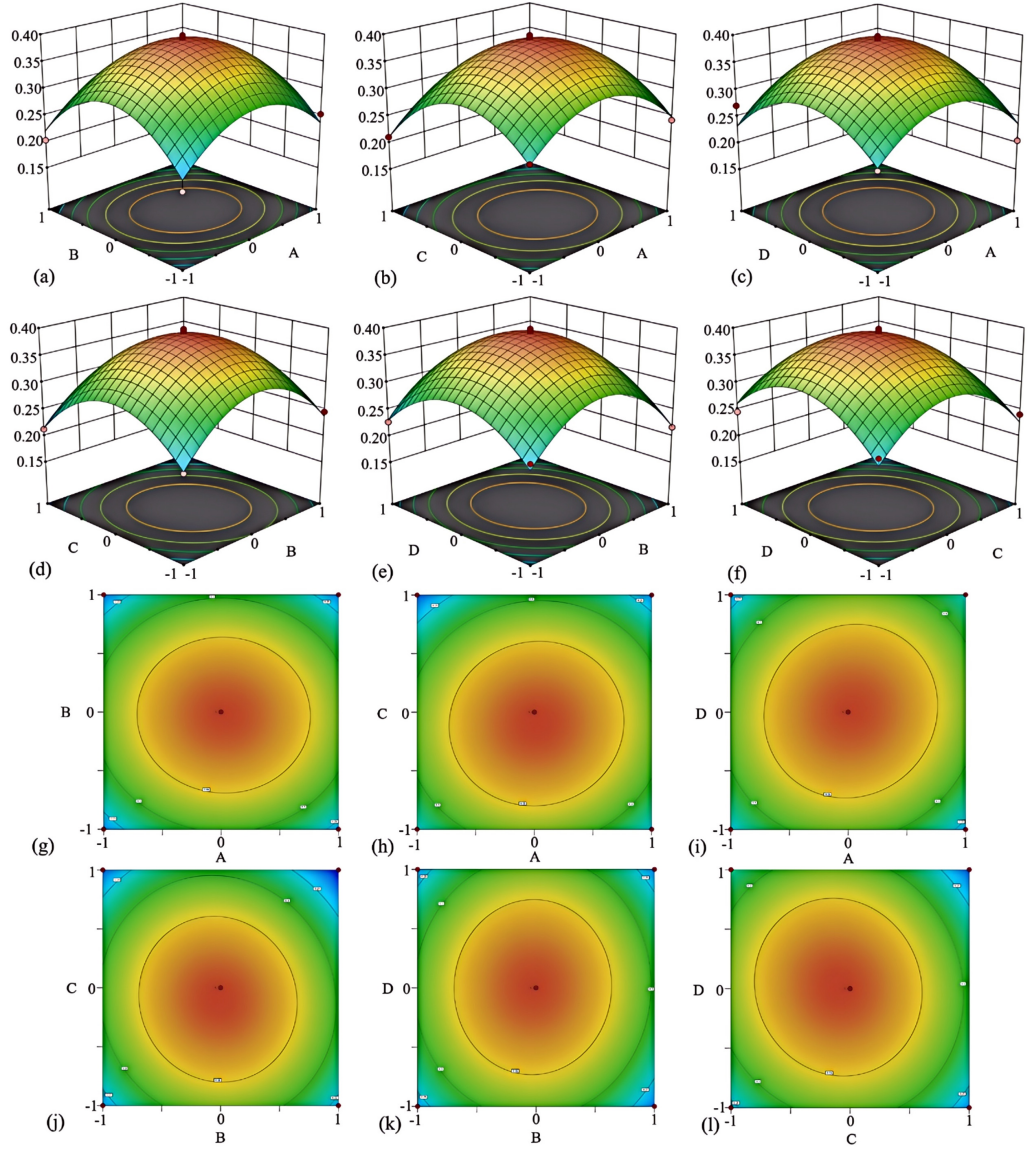
3D interaction diagrams of various factors influencing the RSM of *GJR* and confidence interval results. Interaction between factors A (extraction time) and B (number of extraction) **(a)**; interaction between factors A (extraction time) and C (ethanol concentration) **(b)**; interaction between factors A (extraction time) and D (liquid-solid ratio) **(c)**; interaction between factors B (number of extractio) and C (ethanol concentration) **(d)**; interaction between factors B (number of extractio) and D (liquid-solid ratio) **(e)**; interaction between factors C (ethanol concentration) and D (liquid-solid ratio) **(f)**; **(g–l)** representing the interaction isoclines corresponding to each factor.

The regression equation's first–order term coefficients show each independent variable's influence on the response variable. Results show ethanol concentration most impacts yield, while material–liquid ratio least impacts it. The response surface slope reflects the four factors' influence on the response value, and the surface plot color shows the interaction trend among factors, with darker colors indicating more obvious trends (Figure 4).

Setting two of the four factors at zero levels allows constructing 3D surface and contour plots to observe the interaction effects of the remaining two factors on yield. In the surface plot, steeper surfaces indicate more pronounced factor effects. The contour plot, corresponding to the surface plot, shows stronger interactions between factors when its shape is closer to an ellipse. In conclusion, by integrating the optimal extraction conditions obtained through response surface optimization and simplifying the operation and cost, the extraction conditions were optimized as follows: extraction time of 30 min, two extraction cycles, ethanol concentration of 60%, and a solid-to-liquid ratio of 1:30. Under these conditions, the yield was 3.98%. The extraction conditions optimized by this method are relatively accurate and have practical application value.

To determine the optimal biphasic solvent system, the spectral peak areas of the upper and lower phases of the system were compared using the *K*-value. The *K*-value, calculated by the ratio of the upper phase's spectral peak area (A_U_) to that of the lower phase (A_L_), ranged from 0.5 to 2.0 (29). In this study, 12 solvent systems, comprising *n*-hexane, ethyl acetate, methanol, ethanol, *n*-butanol, petroleum ether, and water, were employed for CCC separation of the target compounds. The *K*-values of these solvent systems are presented in Table 3.

**Table 3:** Partition coefficient of the active ingredient of *G*JR in different solvent systems.

**No**.	**Solvent system**	**v/v/v/v**	***K*_1_**	***K*_2_**	***K*_3_**	***K*_4_**	***K*_5_**	***K*_6_**	***K*_7_**
1	Ethyl acetate/*n*-butanol/water	2.0:1.5:3.0	/	0.63	0.67	0.62	0.68	0.44	0.49
2	Ethyl acetate/*n*-butanol/water	1.0:4.0:5.0	/	0.22	0.22	0.16	0.19	3.29	4.19
3	Ethyl acetate/*n*-butanol/water	1.0:1.0:2.0	/	0.13	0.13	0.09	0.11	0.44	3.57
4	Ethyl acetate/*n*-butanol/water	1.0:2.0:3.0	/	0.37	0.37	0.32	0.38	0.49	6.44
5	Ethyl acetate/ethanol/*n*-butanol/water	9.0:2.0:1.5:16.0	0.61	0.63	0.64	0.71	0.82	0.48	0.46
6	Ethyl acetate/methanol/*n*-butanol/water	9.0:2.0:1.5:16.0	0.59	0.65	0.64	0.71	0.83	0.47	0.44
7	Ethyl acetate/methanol/watert	10.0:1.0:10.0	0.76	0.62	1.27	0.94	0.96	0.32	0.47
8	*N*-hexane/ethyl acetate/methanol/ water	3.0:10.0:3.0:10.0	0.60	0.65	0.64	0.77	0.83	0.48	0.48
9	*N*-hexane/ethyl acetate/methanol/ water	5.0:3.0:3.0:5.0	0.61	0.67	0.68	0.71	0.84	0.19	0.47
10	*N*-hexane/ethyl acetate/ethanol/ water	3.0:10.0:3.0:10.0	0.52	0.87	1.66	0.71	0.96	0.44	0.37
11	*N*-hexane/ethyl acetate/ethanol/ water	1.5:12.0:3.0:10.0	0.66	1.02	1.58	0.73	1.26	1.05	0.94
12	Petroleum ether/ethyl acetate/methanol/water	3.0:7.0:4.0:6.0	0.45	0.36	0.33	0.52	0.24	2.24	1.93

In several of these solvent systems, the value of *K* exceeds 2 or falls below 0.5, resulting in reduced separation efficiency, excessively rapid separation, or unduly slow separation, thereby hindering the attainment of optimal results. The low *K*-value observed for the Ethyl acetate/*n*-butanol/water system suggests that the separation time was insufficient for effective separation. Moreover, compound 1 was undetectable in this solvent system, suggesting inadequate retention of the compound. In the *n*-hexane/ethyl acetate/ethanol/water system, compound 1 exhibited a partition coefficient (*K*_1_) of 0.66, while the other compounds displayed *K*-values approximately equal to 1, which is optimal for separation purposes. Consequently, this solvent system was selected. It effectively resolved compound 1 as well as the other compounds, thereby enhancing the utilization of *GJR*.

**Figure 5:**
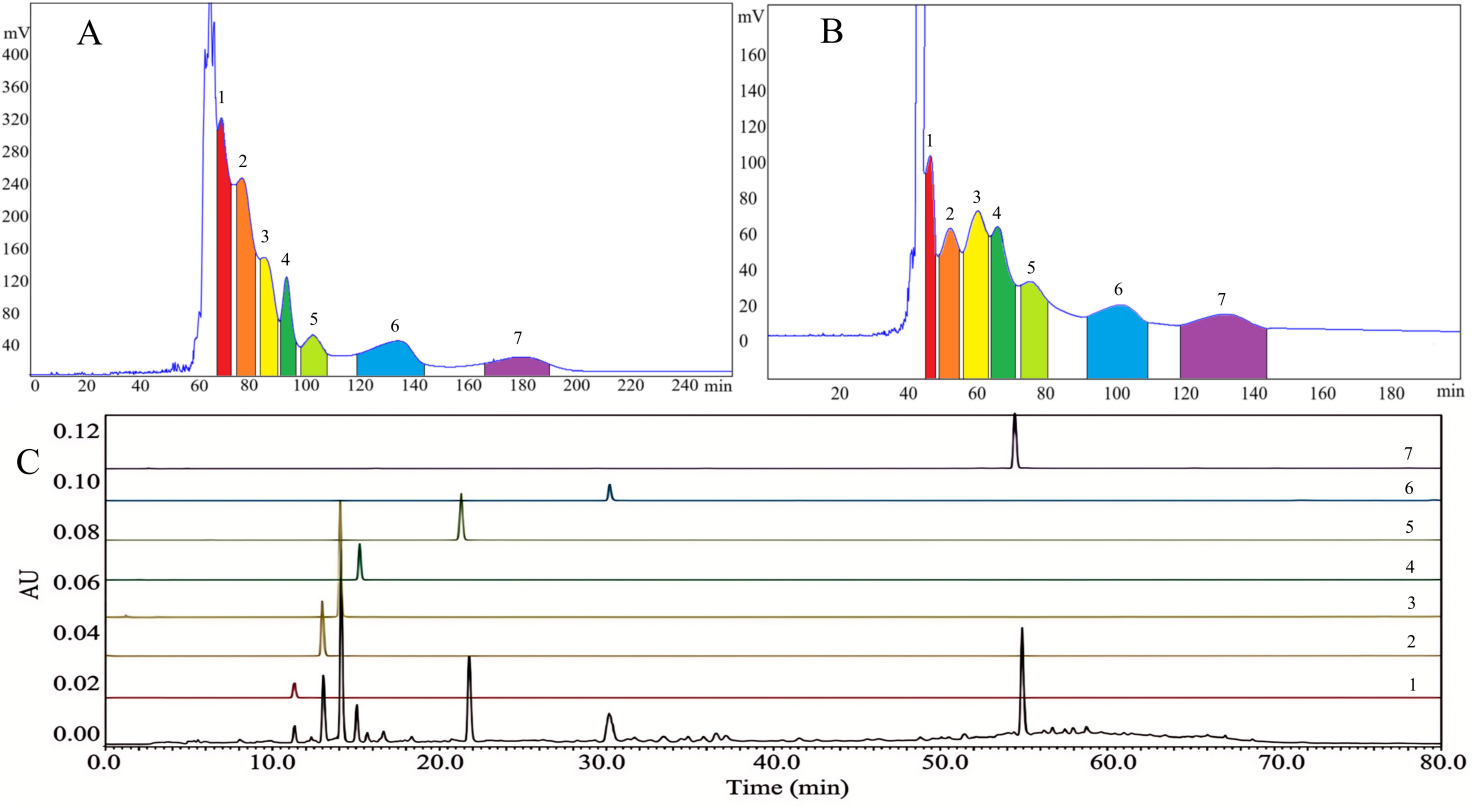
HSCCC **(A)**; and 2D-CCC **(B)**; of the target bioactive compounds in *Gardenia jasminoides* root. HPLC of crude extract obtained from *Gardenia jasminoides* root and the target bioactive compounds **(C)**; (1): Shanziside, (2): Deacetylasperulosidic acid methyl ester, (3): Gardoside, (4): Shanzhiside methyl ester, (5): Mussaenoside acid, (6): Eleutheroside E, (7): 5-Hydroxy-3′,4′-dimethoxyflavone.

The crude extract (300 mg) was fractionated using an *n*-hexane/ethyl acetate/ethanol/water (1.5:12.0:3.0:10.0, v/v/v/v) system, achieving 71.4% retention of the stationary phase and isolating compounds 1–7 (Figure 5A). However, the limited peak capacity of countercurrent chromatography (CCC) resulted in co-elution of compounds with similar *K*-values, thereby hindering peak resolution. To address this issue, we employed the eluate collected after the solvent peak during the first-dimensional CCC for two-dimensional CCC. The resulting two-dimensional chromatogram (Figure 5B) clearly displayed seven distinct peaks, which were further analyzed by HPLC. The findings confirmed the successful separation of compounds 1–7.

**Figure 6:**
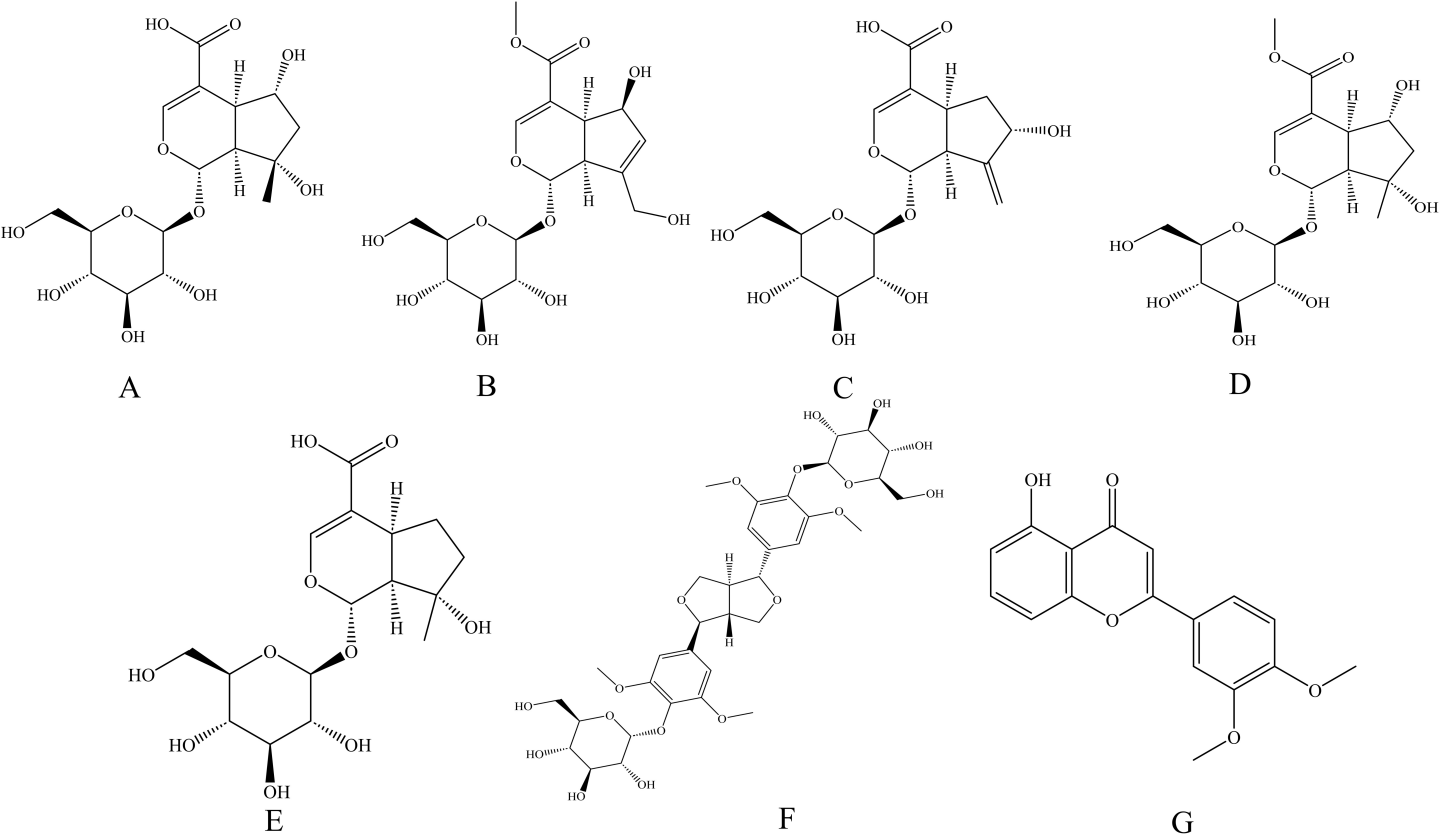
Chemical structures of chemical compounds isolated in this study. **(A)** Shanziside, **(B)** Deacetylasperulosidic acid methyl ester, **(C)** Gardoside, **(D)** Shanzhiside methyl ester, **(E)** Mussaenoside acid, **(F)** Eleutheroside E, **(G)** 5-Hydroxy-3′,4′-dimethoxyflavone.

Each offline 2D-CCC peak fraction was analyzed by HPLC for purity, with the results presented in Figure 5C1–7. The compounds exhibited purities of 96.79, 94.42, 98.45, 95.06, 96.51, 97.88 and 98.57%, respectively, based on the HPLC peak area percentage. The chemical structures of the aforementioned compounds are illustrated in Figures 6A–G. These findings confirmed that the *n*-hexane/ethyl acetate/ethanol/water solvent system at a ratio of 1.5:12.0:3.0:10.0, v/v/v/v was effective for offline 2D-CCC isolation and purification of these seven compounds from *GJR*.

### 3.4 Results of enzyme kinetic analysis

**Figure 7:**
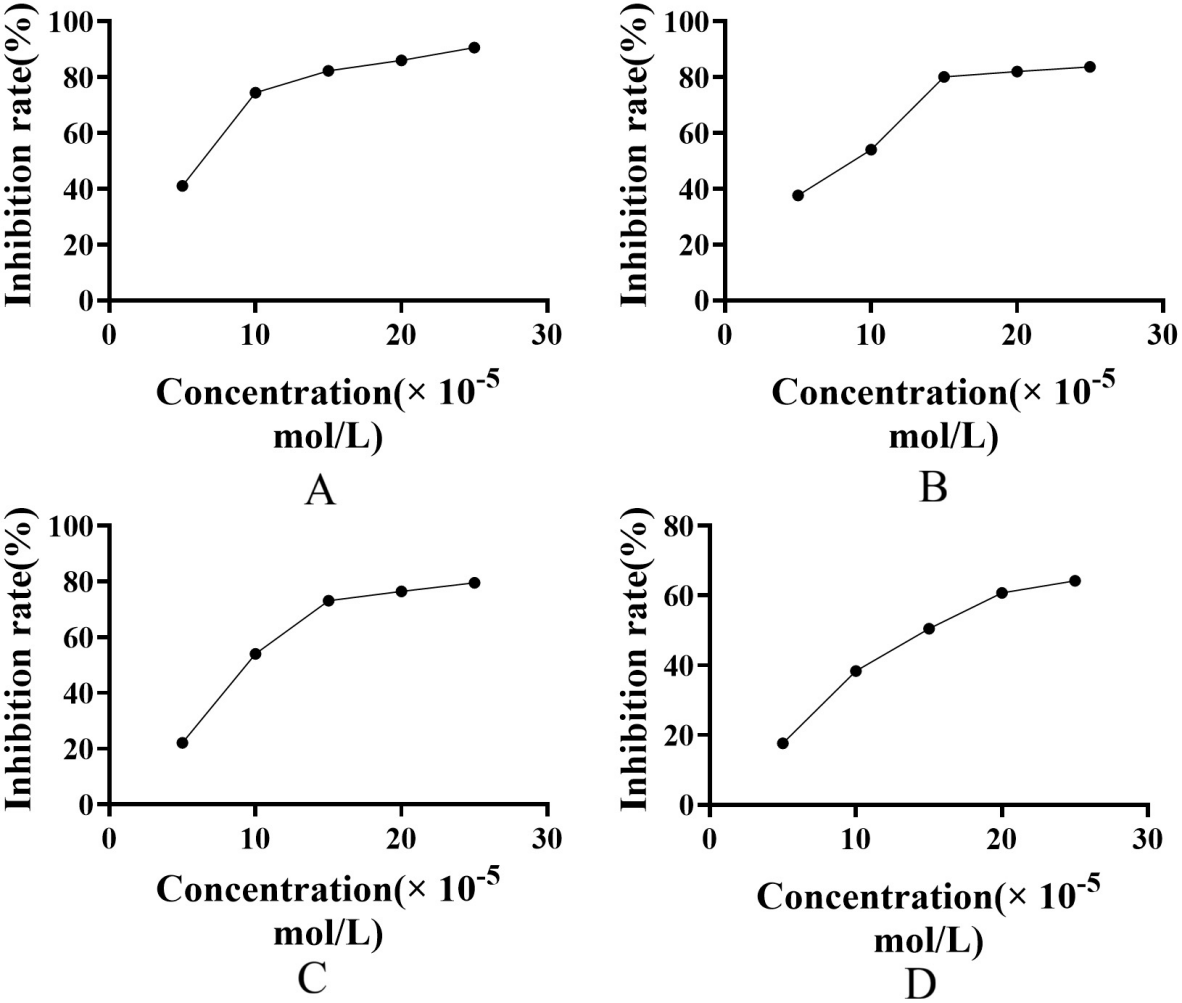
**(A)** Positive control-(5-LOX); **(B)** Positive control-AChE; **(C)** Mussaenoside acid-(5-LOX); **(D)** Mussaenoside acid-AChE.

The AUF experimental results demonstrate that Mussaenoside acid exhibits superior dual-target inhibitory activity. Therefore, enzyme kinetic assays were conducted to evaluate its inhibitory effects on 5-LOX and AChE activities, using Mussaenoside acid as the primary compound of interest. The results are presented in Figure 7.

As shown in the figure, increasing concentrations of Mussaenoside acid led to a significant decrease in enzyme activity and a gradual increase in inhibition rate, which eventually reached a plateau. The IC_50_ values of Mussaenoside acid for 5-LOX and AChE were 4.17 × 10^−5^ and 9.54 × 10^−5^ mol/L, respectively, indicating a stronger inhibitory potency against 5-LOX. For the positive control galantamine, the IC_50_ values were 5.62 × 10^−5^ mol/L against 5-LOX and 7.38 × 10^−5^ mol /L against AChE. These results demonstrate that Mussaenoside acid exhibits effective dual-target inhibition, with slightly greater potency against 5-LOX compared to galantamine (Table 4).

**Table 4:** Results of 5-LOX and AChE inhibition by different monomeric components.

**Sample name**	**Inhibition ratio/%**					**IC_50_**
	**1**	**2**	**3**	**4**	**5**	
	**5-LOX**					
Mussaenoside acid	22.14	54.07	73.14	74.43	79.56	4.17 × 10^−5^
	**AChE**					
	17.66	38.34	50.52	60.78	64.26	9.54 × 10^−5^

**Figure 8:**
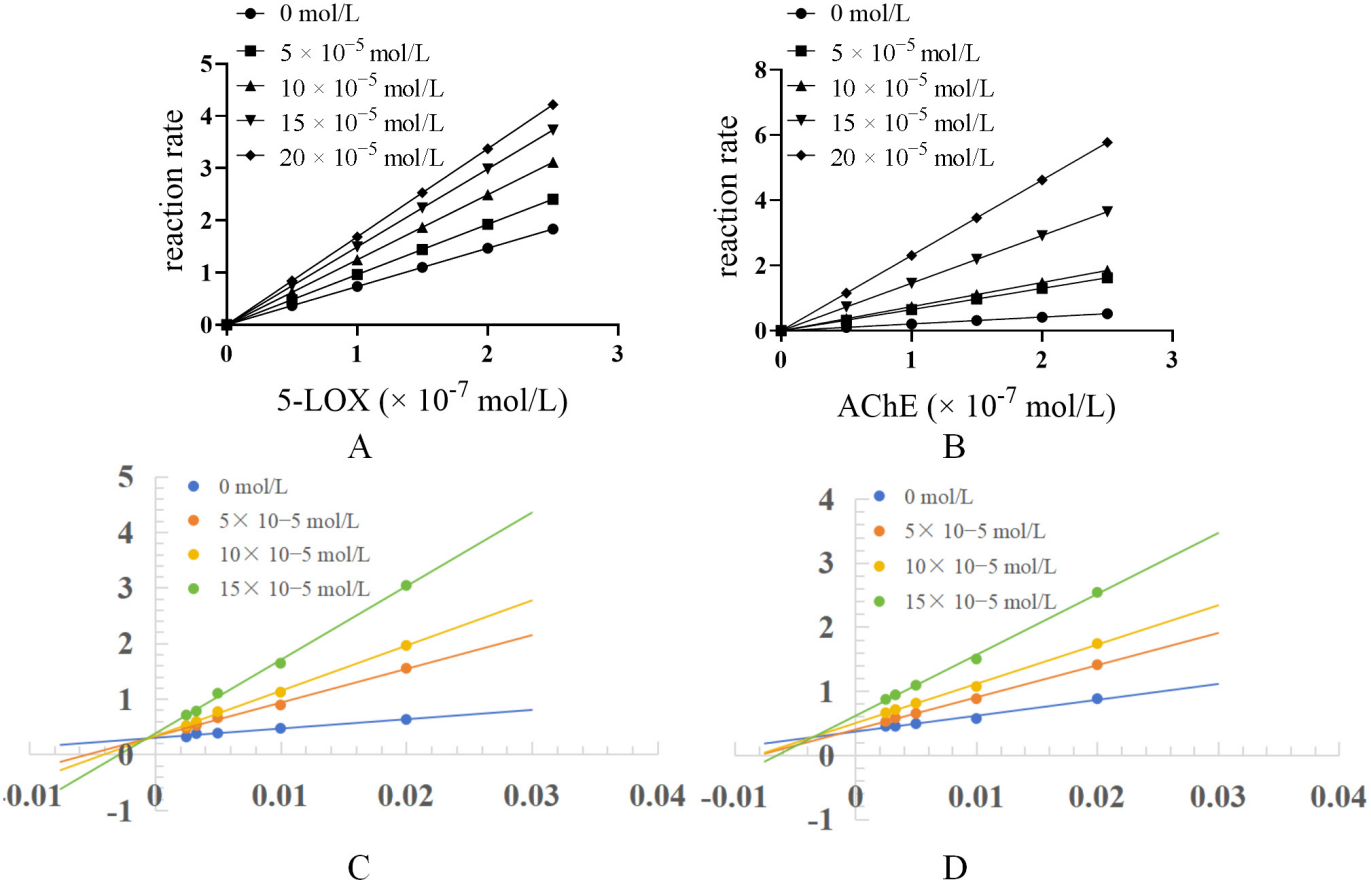
Inhibition kinetics and Lineweaver-Burk plots of 5-LOX and AChE modulated by varying concentrations of Mussaenoside acid. The effect of Mussaenoside acid on enzyme activity: The effect of the compound on 5-LOX activity **(A)**; The effect of the compound on AChE activity **(B)**; Lineweaver-Burk plots of compounds inhibiting 5-LOX activity **(C)**; Lineweaver-Burk plots of compounds inhibiting AChE activity **(D)**.

To assess the reversibility of Mussaenoside acid's inhibition against 5-LOX and AChE, the enzymatic reaction rate was plotted as a function of enzyme concentration (Figures 8A, B). In this study, catalytic activities were measured at varying substrate concentrations and in the presence of different concentrations of Mussaenoside acid to generate Lineweaver-Burk double-reciprocal plots (Figures 8C, D), which were used to determine the inhibition mechanism. The maximum reaction velocity is denoted as V_*max*_, while *v* represents the observed enzymatic reaction rate. [*S*] and [*I*] indicate the concentrations of substrate and inhibitor (Mussaenoside acid), respectively, and K_*i*_ represents the dissociation constant reflecting the binding affinity between the inhibitor and either the free enzyme or the enzyme-substrate complex. A lower K_*i*_ value indicates stronger binding between the inhibitor and the enzyme, thereby conferring greater inhibitory potency.

Figure 8C illustrates that for 5-LOX, the lines corresponding to Mussaenoside acid intersect at a single point on the Y-axis. With increasing compound concentration, the slope of each line increases. V_*m*_ remains largely unchanged, while Km gradually increases, indicating that the inhibition type for 5-LOX is competitive inhibition. The calculated inhibition constants (K_*i*_) is 49.19.

At concentrations below 5 × 10^−5^ mol/L, the lines in the Lineweaver-Burk double reciprocal plot intersect on the y-axis, with the intersection point remaining largely unchanged as the concentration of Mussaenoside acid increases, indicating a competitive inhibition of AChE. In contrast, when the concentration exceeds 10 × 10^−5^ mol/L, a progressive decrease in V_*m*_ and a concurrent increase in K_*m*_ are observed with increasing inhibitor concentration, which is characteristic of mixed-type inhibition. These results suggest that Mussaenoside acid exhibits a transition from competitive to mixed inhibition depending on its concentration (Figure 8D; Table 5).

**Table 5:** Kinetic parameters of mussaenoside acid for the inhibition of 5-LOX and AChE.

**Names of Compounds**	**Sample concentration**	**Michaelis-Menten equation**	***R* ^2^**	***K_*m*_*/*K_*appm*_***	***V_*m*_*/*V_*appm*_***	***K_*i*_***	***K_*is*_***
		**5-LOX**					
Mussaenoside acid	0	1/*v*_1_ = 16.746 × 1/[*S*_1_]+0.2982	0.9805	56.15	3.33	49.19	/
	5	1/*v*_1_ = 26.534 × 1/[*S*_1_]+0.3905	0.9944	85.73	3.22		
	10	1/*v*_1_ = 48.001 × 1/[*S*_1_]+0.2803	0.9933	171.24	3.56		
	15	1/*v*_1_ = 57.69 × 1/[*S*_1_]+0.2913	0.9926	198.04	3.42		
		**AChE**					
	0	1/*v*_2_ = 25.327 × 1/[*S*_2_]+0.3573	0.9845	70.88	2.78	40.2	102.08
	5	1/*v*_2_ = 47.523 × 1/[*S*_2_]+0.4442	0.9958	106.98	2.24		
	10	1/*v*_2_ = 61.3 × 1/[*S*_2_]+0.4978	0.9973	123.14	2.01		/
	15	1/*v*_2_ = 95.103 × 1/[*S*_2_]+0.612	0.9972	153.14	1.60		

### 3.5 Molecular dynamics simulation of active ingredients and target proteins

#### 3.5.1 Molecular docking analysis

**Figure 9:**
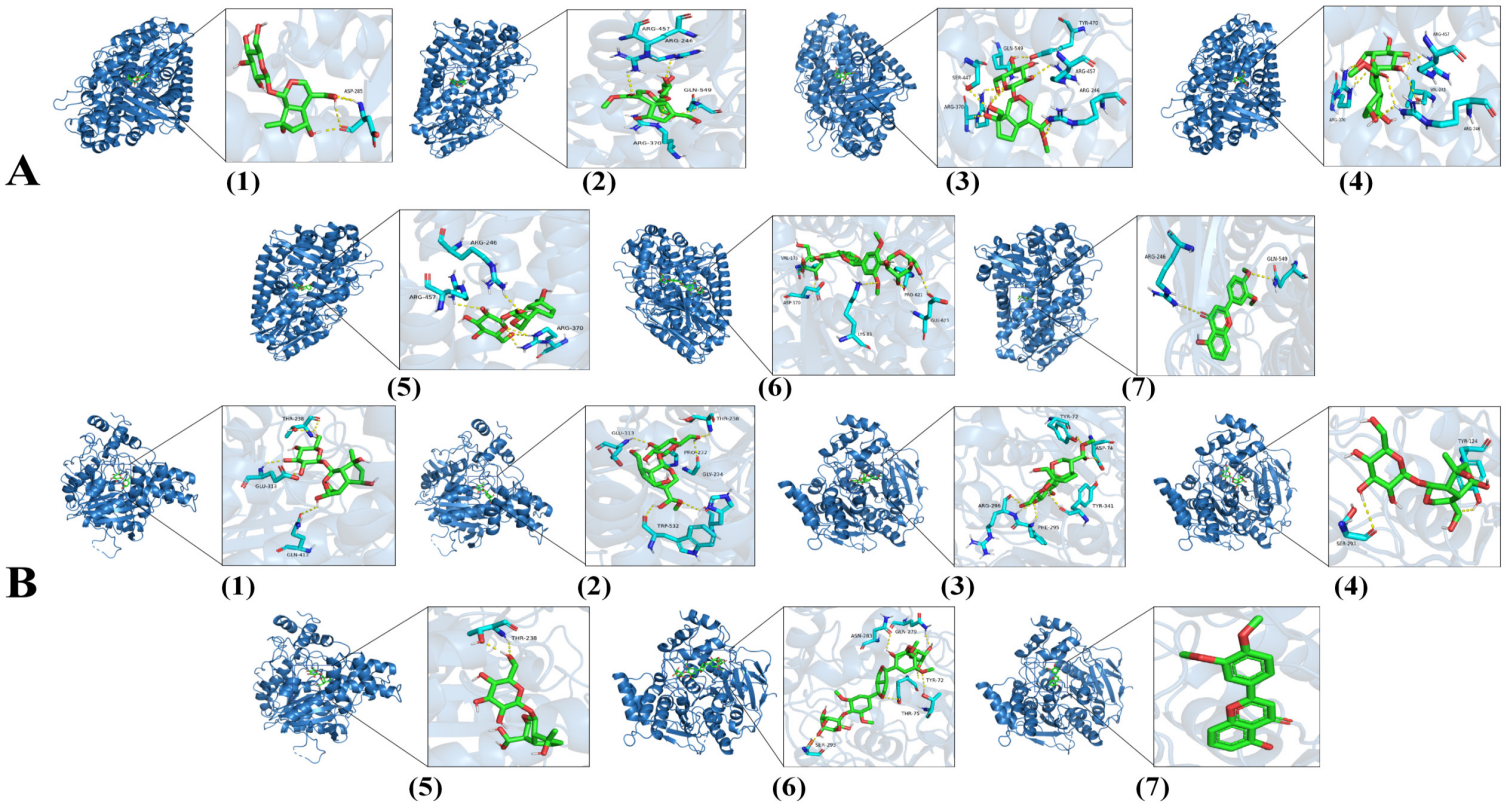
Molecular docking plots of each compound bound to 5-LOX **(A)** and AChE **(B)**; (1): Shanziside, (2): Deacetylasperulosidic acid methyl ester, (3): Gardoside, (4): Shanzhiside methyl ester, (5): Mussaenoside acid, (6): Eleutheroside E, (7): 5-Hydroxy-3′,4′-dimethoxyflavone.

5-LOX and AChE were selected as molecular docking targets to evaluate the binding affinity of Mussaenoside acid through docking scoring, aiming to predict its potential binding sites on these enzymes. The most favorable binding conformation of the system is presented in Figure 9.

The results indicate that Mussaenoside acid binds effectively to both 5-LOX and AChE, with docking binding energies of −7.5 and −7.2 kcal/mol, respectively. In comparison, the positive control galantamine exhibits binding energies of −4.8 kcal/mol for 5-LOX and −6.5 kcal/mol for AChE. These findings demonstrate that Mussaenoside acid has a stronger binding affinity than galantamine toward both target enzymes. Mussaenoside acid engages in hydrogen bonding interactions with the residues ARG246, ARG370, TYR470, and ARG457 within 5-LOX. It also establishes van der Waals interactions with THR366, THR371, LEU448, PHE544, PHE450, ILE454, ALA453, VAL243, LEU244, ILE365, and ALA456, and forms a carbon-hydrogen bond with SER447. Mussaenoside acid forms hydrogen bond interactions with the residues THR238 and GLN413 in AChE, engages in van der Waals interactions with VAL239, GLY234, PRO410, CYS409, LEU536, PRO537, GLU313, THR311, and PRO312, establishes carbon-hydrogen bonds with PRO235 and ASN233, and participates in hydrophobic interactions with HIS405 (Table 6).

**Table 6:** Molecular docking analysis of AChE and 5-LOX with active ingredients.

**Sample name**	**Affinity (kcal/mol)**	**Major interacting amino acid residues**
	**5-LOX**	
Mussaenoside acid	−7.5	ARG246, ARG370, TYR470, ARG457, THR366, THR371, LEU448, PHE544, PHE450, ILE454, ALA453, VAL243, LEU244, ILE365, ALA456, SER447, GLN549
	**AChE**	
	−7.2	THR238, GLN413, VAL239, GLY234, PRO410, CYS409, LEU536, PRO537, GLU313, THR311, PRO312, PRO235, ASN233, ASN533, TRP532, HIS405

#### 3.5.2 Molecular dynamics simulations

**Figure 10:**
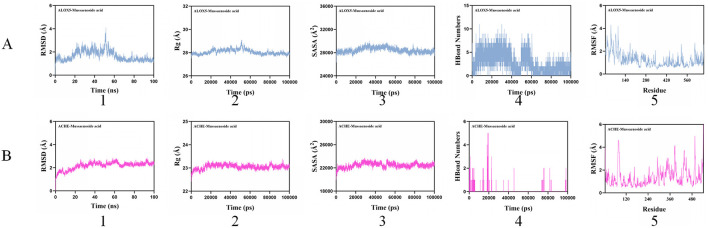
Related indices for molecular dynamics simulation. (1): BMSD, (2): Rg, (3): SASA, (4): HBond Numbers, (5): RMSF. 5-LOX. **(A)** 5-LOX; **(B)** AChE.

Root mean square deviation (RMSD) is a widely used metric for assessing the conformational stability of proteins and ligands, reflecting the extent of atomic displacement relative to their initial positions. Lower RMSD values indicate greater conformational stability. Therefore, RMSD was employed to evaluate the equilibration of the simulation systems. The AChE–Mussaenoside acid complex reached equilibrium after 20 ns and subsequently fluctuated around 2.2 Å. Similarly, the 5-LOX–Mussaenoside acid complex achieved equilibrium after 60 ns, with fluctuations stabilizing near 1.3 Å. The Mussaenoside acid small molecule demonstrates high conformational stability upon binding to the target proteins AChE and 5-LOX. Further analysis revealed that the radius of gyration (Rg) and solvent-accessible surface area (SASA) of the AChE–Mussaenoside acid and 5-LOX–Mussaenoside acid complex systems exhibited slight fluctuations throughout the simulation, indicating conformational changes in the small molecule–target protein complexes. Hydrogen bonds play a crucial role in ligand–protein binding interactions. For the AChE–Mussaenoside acid complex system, the number of hydrogen bonds fluctuated between 0 and 5, with an average of approximately 1 observed during most of the simulation trajectory. In the case of the 5-LOX–Mussaenoside acid complex system, the hydrogen bond count ranged from 0 to 11, with approximately 7 hydrogen bonds maintained on average over time. This suggests that the small molecule–target protein complex exhibits favorable hydrogen bonding interactions. Root mean square fluctuation (RMSF) is a key metric for assessing the flexibility of amino acid residues in proteins. The RMSF values of the AChE–Mussaenoside acid and 5-LOX–Mussaenoside acid complexes are relatively low, predominantly below 4 Å, indicating reduced conformational flexibility and enhanced structural stability (Figure 10).

Considering solvation effects, the stable-state trajectories of the protein–ligand complexes were selected based on a comprehensive analysis of RMSD, Rg, SASA, and interaction energy. The MM-PBSA (Molecular Mechanics–Poisson Boltzmann Surface Area) method was subsequently employed to calculate the binding energy components, as summarized in the Table 7. Specifically, ΔE_ele_ denotes the electrostatic interaction between the small molecule and the protein; ΔE_vdw_ represents the van der Waals interaction; ΔE_pol_ refers to the polar solvation energy, reflecting the electrostatic contribution of solvent effects; ΔE_nonpol_ corresponds to the nonpolar solvation energy, which is associated with hydrophobic interactions; ΔE_MMPBSA_ is defined as the sum of ΔE_ele_, ΔE_vdw_, ΔE_pol_, and ΔE_nonpol_; and ΔG_bind_ is calculated as the sum of ΔE_MMPBSA_ and –TΔS, representing the total binding free energy.

**Table 7:** The binding energy and its constituent components in the stable state (unit: kJ/mol).

**Enzymes**	**ΔE_vdw_**	**ΔE_ele_**	**ΔE_pol_**	**ΔE_nonpol_**	**ΔE_MMPBSA_**	**-TΔS**	**ΔG_bind_**
5-LOX	−106.473 ± 0.652	−54.426 ± 2.47	125.473 ± 3.697	−16.631 ± 0.126	−54.263 ± 1.577	19.0.53 ± 2.127	−31.797 ± 1.082
AChE	−177.763 ± 2.441	−40.564 ± 1.83	133.56 ± 3.382	−25.463 ± 0.178	−120.273 ± 1.866	24.542 ± 3.156	−89.213 ± 4.35

ΔGbind = ΔEvdw+ΔEele+ΔEpol+ΔEnonpol-TΔS.

Analysis of Table 7 reveals that the ΔEMMPBSA binding energy of mussaenoside acid with 5-LOX is higher than that with AChE, indicating stronger binding affinity toward 5-LOX compared to AChE. The negative electrostatic interaction values between mussaenoside acid and both enzymes suggest that electrostatic interactions favorably contribute to protein binding. Furthermore, van der Waals forces are stronger than electrostatic interactions in the complexation with both enzymes, indicating that van der Waals forces dominate the binding energy composition, whereas electrostatic interactions play a supporting role.

## 4 Discussion

Alzheimer's disease (AD) is a progressive neurodegenerative disorder that affects the central nervous system and is characterized by gradual cognitive decline and behavioral impairment (30). Unfortunately, to date, only a limited number of drugs have received FDA approval for the treatment of AD, including galantamine, donepezil, and risperidone. These medications function by enhancing acetylcholine levels in the brain. However, these agents exhibit suboptimal efficacy and are associated with significant adverse effects, such as hepatotoxicity (31, 32). According to published studies, approximately 189 plant-derived drugs have been licensed for clinical use (33–35), reducing the risk of certain chronic diseases, particularly neurodegenerative disorders, with a specific emphasis on AD (36). Traditional Chinese medicine (TCM) possesses an extensive history in AD treatment and serves as a promising alternative owing to its minimized toxic side effects (37, 38). Therefore, it is imperative to systematically screen and identify the anti-AD components in herbal medicines for further development and utilization.

In this study, seven compounds were identified to bind specifically to 5-LOX and AChE, showing potential enzyme inhibitory effects. The binding degree was calculated using a specific equation. Overall, the seven active components exhibited effective dual-target inhibition with increasing enzyme concentration, among them, mussaenoside acid exhibits the most potent effect. AUF-LC-MS is employed for the screening and identification of compounds that exhibit specific biological activities, thereby facilitating the elucidation of the mechanisms of action of natural products (39). It enables rapid screening and precise identification of active ingredients in complex samples, and can be integrated with techniques such as molecular docking, kinetic simulation, and enzyme reaction kinetics to investigate the mechanism of action of active ingredients more comprehensively (40).

Subsequently, network pharmacology identified a total of 90 shared targets associated with diseases and active ingredients. Through GO enrichment analysis and KEGG pathway enrichment analysis, the results revealed potential targets involved in biological processes such as inflammatory response and cell cycle regulation. These processes are closely linked to the pathogenesis of AD, the targets identified through network analysis are consistent with those derived from empirical data, thereby reinforcing the theoretical foundation of the multi-target paradigm.

In addition, seven active substances were isolated from *GJR* extracts using CCC. However, the limited peak capacity of CCC resulted in co-elution of compounds with similar *K* values, thereby affecting peak resolution. To address this issue, offline 2D-CCC technology was employed, successfully separating the seven compounds with purities exceeding 90%.

Molecular docking and dynamics simulations were employed to predict and analyze the molecular interactions and dynamic behaviors. Among 5-LOX inhibitors, mussaenoside acid exhibits a calculated binding energy of −7.5 kcal/mol. The main acting residues were ARG246, ARG370, TYR470, ARG457, THR366, THR371, LEU448, PHE544, PHE450, ILE454, ALA453, VAL243, LEU244, ILE365, and ALA456, SER447. Among the AChE inhibitors, mussaenoside acid exhibits a calculated binding affinity of −7.2 kcal/mol. Key interacting residues included THR238, GLN413, VAL239, GLY234, PRO410, CYS409, LEU536, PRO537, GLU313, THR311, PRO312, PRO235, ASN233, HIS405 (Table 6).

These findings underscore the therapeutic potential of *GJR* active ingredients in the management of AD, elucidating the interaction between these drug components and the disease mechanism. The seven promising active ingredients identified through network pharmacology align with those detected via AUF-LC-MS screening, targeting a broader spectrum of associated pathways. This congruence implies that *GJR* may serve as a promising agent for AD treatment. Furthermore, the results of the network pharmacology analysis validate the experimental accuracy, thereby reinforcing the comprehensiveness and robustness of the overall experimental design.

## 5 Conclusion

Natural products have long served as an essential resource for drug discovery, drawing sustained interest from researchers worldwide. This study focused on the anti-AD potential of GJR by evaluating its inhibitory activity against 5-LOX and AChE. We developed a three-tiered evaluation framework encompassing *in vitro* screening, computational simulation, and enzymatic kinetic assays. This integrated strategy enabled the rapid identification and structural characterization of active compounds, elucidation of their binding sites, and mechanistic insights into their inhibitory interactions with 5-LOX and AChE. Collectively, the approach facilitated the systematic screening, isolation, and efficacy assessment of bioactive constituents.

In this study, AUF-LC-MS was employed to screen seven potential dual-target inhibitors from GJR extracts by targeting 5-LOX and AChE. Network pharmacology was utilized to predict the therapeutic targets of active ingredients in GJR for Alzheimer's disease. Optimal extraction conditions were established as follows: extraction time of 0.5 h, extraction frequency of twice, ethanol concentration of 60%, a liquid–solid ratio of 30 ml/g, and a yield of 3.98%. Seven high-purity monomer compounds were successfully isolated via off-line two-dimensional counter-current chromatography (2D-CCC), all with purities exceeding 90%. The enzymatic assay results demonstrate that mussaenoside acid exerts reversible inhibition against 5-lipoxygenase and mixed-type inhibition against acetylcholinesterase. The half-maximal inhibitory concentration results demonstrate that mussaenoside acid exhibits potent dual-target inhibitory activity, with slightly greater potency against 5-LOX compared to galantamine. Molecular docking and molecular dynamics simulations were employed to investigate the binding mechanisms of mussaenoside acid with the target enzymes. The results demonstrated that the inhibitors could spontaneously interact with 5-LOX and AChE, forming stable complex systems.

In conclusion, our systematic investigation contributes to improving the resource utilization and scientific understanding of *GJR*, laying a solid foundation for further pharmacological exploration. However, the current findings are limited to *in vitro* studies. Future research will focus on validating the anti-AD efficacy of *GJR* through animal models, with the aim of providing more comprehensive theoretical and empirical support for its clinical application.

## Data Availability

The raw data supporting the conclusions of this article will be made available by the authors, without undue reservation.
